# Structured Back Focal Plane Interferometry (SBFPI)

**DOI:** 10.1038/s41598-019-56199-z

**Published:** 2019-12-30

**Authors:** Avinash Upadhya, Yujie Zheng, Li Li, Woei Ming Lee

**Affiliations:** 1 Research School of Electrical, Energy, and Materials Engineering, College of Engineering and Computer Science, The Australian National University, Canberra, ACT 2601 Australia; 2 The State Key Laboratory of Optoelectronic Materials and Technologies, Sun Yat-sen University, Guangzhou, 510275 China; 3 The ARC Centre of Excellence in Advanced Molecular Imaging, The Australian National University, Canberra, ACT 2601 Australia

**Keywords:** Engineering, Optics and photonics

## Abstract

Back focal plane interferometry (BFPI) is one of the most straightforward and powerful methods for achieving sub-nanometer particle tracking precision at high speed (MHz). BFPI faces technical challenges that prohibit tunable expansion of linear detection range with minimal loss to sensitivity, while maintaining robustness against optical aberrations. In this paper, we devise a tunable BFPI combining a structured beam (conical wavefront) and structured detection (annular quadrant photodiode). This technique, which we termed Structured Back Focal Plane Interferometry (SBFPI), possesses three key novelties namely: extended tracking range, low loss in sensitivity, and resilience to spatial aberrations. Most importantly, the conical wavefront beam preserves the axial Gouy phase shift and lateral beam waist that can then be harnessed in a conventional BFPI system. Through a series of experimental results, we were able to tune detection sensitivity and detection range over the SBFPI parameter space. We also identified a figure of merit based on the experimental optimum that allows us to identify optimal SBPFI configurations that balance both range and sensitivity. In addition, we also studied the resilience of SBFPI against asymmetric spatial aberrations (astigmatism of up to 0.8 λ) along the lateral directions. The simplicity and elegance of SBFPI will accelerate its dissemination to many associated fields in optical detection, interferometry and force spectroscopy.

## Introduction

**Figure 1:**
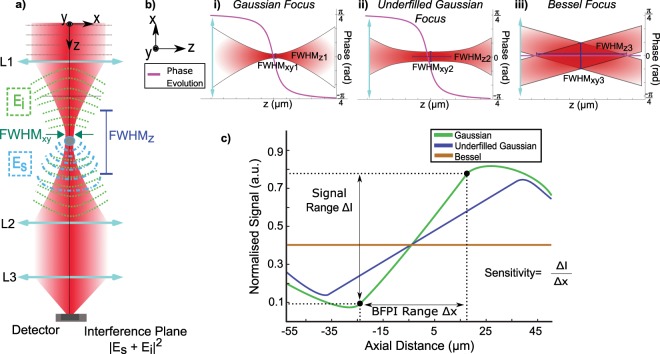
(**a**) A simplified illustration of BFPI showing how the unscattered component E_i_ and the scattered light E_s_ interfere at the interference plane to produce a position-dependent intensity. (**b**) Illustrating intensity profiles for three beam candidates for BFPI, (i) standard Gaussian focus, (ii) Underfilled/Low NA Gaussian focus, and (iii) Bessel Beam. These beams are illustrated with their axial Full Width Half Maximum (FWHM_z_), their beam waist (FWHM_xy_), and their axial phase progression (purple). In (**c**) we illustrate the corresponding BFPI signal calibration curves for the beams discussed in (**b**).

Interferometric signals at the back focal plane of a microscope objective lens of any numerical aperture (NA) provide unique insight into the scattering dynamics of microscopic particles. Back focal plane interferometry (BFPI)^1–3^ can be seen as an ubiquitous on-axis interference effect that has been heavily used in force microscopy and spectroscopy. When the focus of a laser beam is brought into contact with a particle of interest, a proportion of the light is scattered. BFPI rests on the measurable interference signal from this process at the back focal plane of an objective lens, resulting in a particle position-dependent intensity I_BFP_^4^. A single BFPI pattern is a time-averaged intensity pattern (I_BFP_) from the coherent interference between the scattered electric field of a particle E_s_ and the propagating incident electric field E_i_ along the axial direction (z), as illustrated in Fig. (1a). The positions of the particle are directly correlated to relative changes in this first order interference pattern of the particle positioned a small distance away from the focus of a Gaussian beam.

BFPI^4–9^ has advantages over image based particle tracking techniques^10,11^ because it captures all positional information with a single high speed detector unit (quadrant photodiode detector (QPD) or position sensitive detector) in a single shot. There are two key parameters crucial to displacement detection: range (the spatial extent over which particle displacement can be measured)^5,8^ and sensitivity (the concomitant change in the BFPI signal for a given change in particle position)^12,13^. These two parameters are dependent on three parameters of the detector beam: lateral intensity, axial intensity and Gouy phase^14^. The size of the microscopic object being measured has an effect on the scattering signal and hence the I_BFP_ in lateral (x, y) and axial (z) direction and has been investigated previously in^2^, but we hold this constant as we are focused on the properties of the beam itself. The relationship between linearity over the detection range and detection sensitivity of BFPI is tied to the properties of the detection beam as described by Pralle *et al*.^1^ in Eqs. (1) to (3) below.

1 $${I}_{{\rm{BFP}}}\approx {{\rm{c}}}_{{\rm{s}}}\,{\rm{\varepsilon }}\,{\rm{Re}}\{{{\rm{E}}}_{{\rm{i}}}{{\rm{E}}}_{{\rm{s}}}\}$$

2 $$\frac{{I}_{{\rm{B}}{\rm{F}}{\rm{P}}{\rm{x}}{\rm{y}}}}{{I}_{{\rm{t}}{\rm{o}}{\rm{t}}{\rm{a}}{\rm{l}}}}({\rm{x}})\propto (\frac{x}{FWH{M}_{xy}^{3}}){{\rm{e}}}^{-{({\rm{x}}/FWH{M}_{xy})}^{2}}$$

3 $$\frac{{I}_{{\rm{B}}{\rm{F}}{\rm{P}}{\rm{z}}}}{{I}_{{\rm{t}}{\rm{o}}{\rm{t}}{\rm{a}}{\rm{l}}}}(z)\propto (\frac{1}{FWH{M}_{xy}^{2}\,}){(1+{(\frac{{\rm{z}}}{FWH{M}_{z}})}^{2})}^{-1/2}\,\sin (\alpha ),\,where\,\alpha ={\tan }^{-1}(z/FWH{M}_{z})$$

where c_s_ is the speed of light of the medium, ε is the dielectric constant of the sphere, FWHM_xy_ is the beam waist at the focus, x, y, and z are the lateral and axial positions respectively, and FWHM_z_ refers to the Rayleigh range that is experimentally quantified by the full width half maximum (FWHM) of the axial intensity distribution. E_i_ is the incident field and E_s_ scattered field that together interfere to produce the signal of interest at the back focal plane I_BFP_. The Gouy phase, −π/4 < α < π/4, is the phase shift between the scattered (E_s_) and unscattered light (E_i_) in the far-field used to detect particle displacements.

As described in Eq. (1), the BFPI signal is a result of the interference between the scattered and unscattered components of light. Due to the interdependence of the 3D beam intensity profile and the effects of Gouy phase for particle position tracking there are limited techniques that can readily tune the BFPI signal using Gaussian beams, especially since a single QPD is used to measure both lateral and axial position simultaneously^15^. The restrictions on range can primarily be attributed to the focusing properties of Gaussian beams through an objective lens with fixed NA^2,16^, but the sensitivity is another matter. Equations (2) and (3)^1^ give the sensitivity of the lateral $(\frac{{I}_{{\rm{BFPx}}}}{{I}_{{\rm{total}}}})$ and axial $(\frac{{I}_{{\rm{BFPz}}}}{{I}_{{\rm{total}}}})$ signals and from these, we can see that both the lateral and axial signals are dependent on the lateral beam waist (FWHM_*xy*_) at the focus. The lateral and axial sensitivities are proportional to the inverse cube and the inverse square respectively. In addition to FWHM_xy_, the axial signal sensitivity $\frac{{I}_{{\rm{BFP}}}}{{I}_{{\rm{total}}}}({\rm{z}})$ is additionally dependent on the Gouy phase α.

Figure 1b(i) illustrates the condition for a standard Gaussian beam. While a Gaussian focus has a relatively short FWHM_z_, it possesses the critical Gouy phase shift (α) and small FWHM_xy_ necessary for axial BFPI $(\frac{{I}_{{\rm{BFPz}}}}{{I}_{{\rm{total}}}})$. Using Eqs. (2) and (3), these factors imply a BPFI signal that has shorter axial range, but high sensitivity. Based on Eq. (3), we see that it is possible to increase the axial detection range only if we can produce a beam that possesses a longer axial full width half maximum (FWHM_z_) while retaining the Gouy phase.

In practice, there are two straightforward ways to achieve a longer FWHM_z_ for increasing axial BFPI range which are illustrated in Fig. 1b(ii,iii). Firstly, one can underfill the beam onto the objective lens as in Fig. 1b(ii). Practically this is done by introducing an iris prior to the objective, or by introducing the detector beam from a low NA condenser lens instead of a high NA trapping objective lens as described by Martinez *et al*.^3^. It has been shown that the detection beam through a lower NA objective extends the position detection range because both lateral and axial intensity distribution of the detector beam are increased, while the Gouy phase necessary for axial BFPI tracking is retained. Unfortunately, this 3D expansion of the FWHM_xy_ and FWHM_z_ means that axial sensitivity is reduced in accordance with Eq. (3) ^2–4,16^. A simple substitution of a 2-fold increase in FWHM_xy_ for $\frac{{I}_{{\rm{BFPx}}}}{{I}_{{\rm{total}}}}$ and $\frac{{I}_{{\rm{BFPz}}}}{{I}_{{\rm{total}}}}$ in Eqs. (2) and (3) resulted in the reduction of the axial and lateral sensitivity by over 800% and 400% respectively^1,3^. On a side note, other studies have shown that sensitivity decreases but range increases with smaller detection angle (lens with lower NA) and vice versa^17^. Hence, the intuitive method to increase the range in the lateral and axial range of BFPI, which is to expand and elongate the intensity distribution in all directions by underfilling the objective with the illuminating Gaussian beam, becomes much less desirable^3^.

Secondly, a Bessel beam can be considered another candidate which is well-known to achieve extended depth of field, as shown in Fig. 1b(iii). However, the Bessel beam displays minimal Gouy phase shift as it is a result of the interference of annular converging oblique plane waves^18^. This means it does not present the characteristic Gouy phase shift, severely affecting $\frac{{I}_{{\rm{BFPz}}}}{{I}_{{\rm{total}}}}$. In reality, the axial phase distribution becomes smaller in magnitude^18^ and progresses much more slowly compared to a Gaussian. Although the Bessel beam may display a longer FWHM_z_, the lack of Gouy phase shift means that it cannot be used for BFPI.

To summarize and compare the beams discussed above, we illustrate their effects on the axial BFPI signal curves in Fig. 1(c). The Gaussian focus (green) has a relatively short range (shorter linear region) but a higher gradient (sensitivity). A lower NA Gaussian focus (blue) has the effect of extending the range (longer linear region), but the extended beam waist results in a significantly lower gradient (lower sensitivity). These illustrate the necessity of extending the FWHM_z_ while maintaining FWHM_xy_. In the case of the Bessel beam, the FWHM_z_ is significantly extended but there is minimal Gouy phase shift. The brown curve in Fig. 1(c) depicts the expected position dependent intensity change that could be visible in the BFPI signal. The ideal beam that satisfies Eqs. (2) and (3) with large range and minimal loss of sensitivity is a beam possessing a Gouy phase anomaly, minimal FWHM_xy_, and maximal FWHM_z_.

There are numerous benefits of increasing the detection range of BFPI. A direct extension of the tracking range in all directions (∆x, ∆y, ∆z) could possibly benefit the measurable range of optical trapping forces (F = k ∆x)^19^, since a greater ∆x increases the measurable F. Also this form of BFPI could assist in direct tracking of non-spherical objects such as nanorods and nanowires^20^, providing high-precision, high speed, single shot measurements of position in all three dimensions. There are also low numerical aperture 3D optical trapping geometries^21^ that would benefit from a tunable BFPI technique. Previous attempts to improve the range of BFPI tracking include the introduction of scanning beams^17^, complex position calibration^17^, or reduction of the NA of the lenses^3^. Friedrich and Rohrbach^17^ employed a detuned (acoustic-tuned) trapping laser beam with an adjustable axial control so as to extend the linear detection range, requiring a second optical source. Deufel and Wang^22^ used a DNA sequence with a known unzipping force-extension characteristic to identify the axial displacements. On the detection side, proposed improvements include moving the detector to the image plane^8^, as well as introducing a stop at the back focal plane of the detection lens^23^.

In this paper, we extend the displacement measurement range by drawing upon new structured beams and structured detectors, which we term Structured Back Focal Plane Interferometry (SBFPI). We generated an elongated axial beam intensity profile (FWHM_z_) by imparting a conical wavefront onto a Gaussian beam, which we refer to as a Conical Wavefront Gaussian Beam (CWGB). We stress that the CWGB is not a Quasi Bessel Beam (QBB). QBBs require an annular intensity projected at the back focal plane of an objective lens^18^. Instead, the CWGB uses a conical phase-only element placed at the back focal plane of the objective. We demonstrate that this CWGB produces an elongated axial beam intensity profile (FWHM_z_) that is comparable to a QBB. More importantly, CWGBs retain the important axial phase shift (Gouy phase jump of π/2) required to maintain high axial sensitivity $\frac{{I}_{{\rm{BFPz}}}}{{I}_{{\rm{total}}}}$^1^. We then combined the CWGB with a structured detection method through use of an annular QPD (AQPD), as proposed in^23^ where the central portion of a traditional QPD is blocked. Existing BPFI systems are sensitive to spatial aberrations^24^, and so we also show that this combination of CWGB with the AQPD not only increases detection range but is robust against spatial aberrations.

### Conical wavefront beam: gouy phase, lateral and axial intensities

To test our hypothesis in the previous section, we perform numerical simulations to study the FWHM_xy_, FWHM_z_, and Gouy phase effects for two types of beams known to extend the FWHM_z_ while maintaining a narrow FWHM_xy_, namely; QBBs and CWGB. The QBB is engineered by placing a simple binary annular aperture at the back focal plane of an objective lens^25^. A CWGB beam is generated by placing a conical phase element at the back focal plane of the objective. Our BPFI setup is based on Martinez *et al*.^3^ where the tracking beam is introduced from a lower NA objective lens. This approach is flexible because it is easily implemented and decoupled from the need to use a high NA lens.

**Figure 2:**
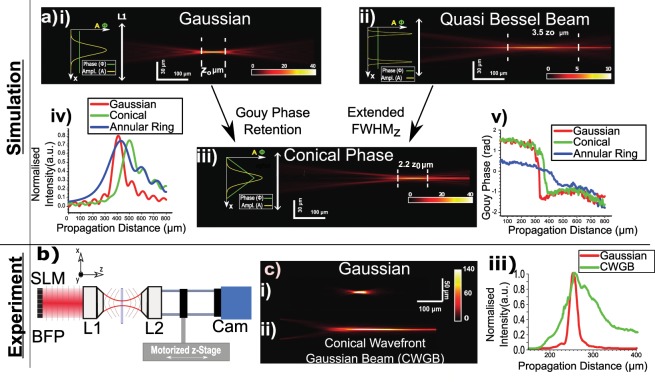
Extending depth of focus with conical phase. (**a**)(i), (ii), and (iii) illustrate the simulated 2D beam intensity profiles for a flat phase Gaussian beam, a Quasi-Bessel Beam, and a Conical Wavefront Gaussian Beam (CWGB). The CWGB combines useful properties of the Gaussian and the Quasi-Bessel Beam - a moderate improvement in FWHM_z_ with the same intensity scale as the Gaussian. (**a**)(iv) and (v) present the normalized intensity profile, and the axial phase evolution against propagation distance for the Gaussian, CWGB, and Quasi-Bessel Beam. This shows the minimal Gouy phase shift of the Quasi-Bessel beam. (**b**) Shows the setup used to measure the beam intensity profile, with the spatial light modulator (SLM) at the back focal plane of L1. (**c**) Shows the experimentally obtained beam profiles of (i) Gaussian, and (ii) CWGB in identical conditions. This shows the elongation of the intensity for the CWGB. Finally, (iii) shows the normalized axial beam intensity profile from (i) and (ii), which confirm that the Gaussian has a shorter FWHM_z_ than the CWGB.

In the simulation model, we used a thin lens model (NA~0.1) that matches our BFPI implementation as described by Martinez *et al*.^3^. Although the thin lens model does not accurately account for non-paraxial rays of modern objective lenses, it does provide a qualitative prediction of the FWHM_xy_, FWHM_z_ and the expected Gouy phase evolution of a converging lens. We first calculate a propagating electric field distribution E(x, y, z) using step split two-dimensional discrete Fourier transformation of a Gaussian beam with a quadratic phase factor of a positive lens, $\exp (-i\frac{k}{2f}{r}^{2})$, where ${r}^{2}={x}^{2}+{y}^{2}$ and *f* is the focal length of the lens, at different propagating distances. Figure 2(a) shows the 2D intensity profiles generated from this simulation for three candidates: (i) a standard Gaussian beam, (ii) a QBB^25^, and (iii) a CWGB. Figure 2(a)(iv) presents the normalized axial beam intensity profiles, while v) plots the Gouy phase evolution against axial distance.

Figure 2(a)(i) shows the intensity cross-section of a Gaussian beam undergoing focusing with a depth of focus, FWHM_z_, and the presence of the Gouy phase jump can be seen in Fig. 2(a)(v). The Gaussian beam is the standard beam used in BFPI and serves as a standard of comparison. Next, in Fig. 2(a)(ii), we modeled the generation of a QBB by using a standard binary annular ring placed at the back-pupil plane of a converging lens. The annular ring with thickness around 11% of the diameter of the pupil of the lens creates a ring illumination on the positive lens. This resulting beam, also known as a Bessel-Gaussian field, exhibits an elongated optical focus of approximately 3.5 FWHM_z_ with minimal changes of the beam waist at the focus (<10%). However, this significantly reduces the intensity by over a factor of 4, which can present issues with Signal-to-Noise Ratio (SNR) as well as the range of detection. In addition, it can be seen in Fig. 2(a)(v) that the Gouy phase jump is much smaller and slower in progression than the other candidates This has negative implications according to Eq. (2), and so we deem this QBB field not suitable for use in BFPI.

Figure 2(a)(iii) shows the propagation dynamics of a conical wavefront delay with a given conic constant imposed onto a Gaussian beam (the CWGB). This is akin to placing a conical element at the back-pupil plane of the objective. The intuitive reason for this wavefront is that it would generate a linearly radial phase delay across the width of the beam which can also result in an elongated focal depth. The conical phase distribution is given by $\varnothing \,=2\,\pi \,r/{d}_{c}$, where r is the unit circle and d_c_ is radial period of the conical phase. Similar to spherical aberration, the marginal rays and paraxial rays focus at different positions along the principal optical axis leading to an elongated focal depth. The numerical simulation confirms this effect using a conical phase constant, d_c_ of 0.12. The beam is elongated by over 2.2 z_o_ with minimal loss of intensity or increase of lateral beam waist (within 10%). Moreover, the conical phase retains the rate of phase change along the axial direction as seen in the Gouy phase jump present in Fig. 2(a)(v). The model demonstrates that the addition of a conical element provides a controllable means to vary the linear range and sensitivity for BFPI displacement measurements. The conical wavefront Gaussian beam (CWGB) with extended axial intensity retains two key properties for effective BFPI; namely, it retains the Gouy phase and experiences minimal change of lateral beam waist over the axial direction.

We next constructed an experimental setup as shown in Fig. 2(b) to investigate the extended 2D intensity profile in a CWGB relative to a Gaussian beam and so verify our simulation prediction. The experimental setup shows a spatial light modulator (SLM; Meadowlark Optics 512 × 512) placed at the back focal plane of the lens L1. The resultant detection beam (635 nm fibre coupled diode laser, iFlex 1000) is introduced from the condenser lens L1 (Mag = 10x , NA = 0.30, air) before being collected by the higher NA trapping objective lens L2 (Mag = 40x, NA = 0.80, air), where NA refers to the numerical aperture. L2 and the camera are fixed in position and placed on a motorized stage. The purpose of this part of the setup is to image the focus of the detection beam directly onto the camera, and by moving the motorized stage it is possible to image across the depth of focus of the detection beam. In Fig. 2(c)(i,ii) we show the 2D cross-section intensity plot of the Gaussian beam and CWGB. To quantify and compare the axial intensity distribution, we plotted the normalized 1D axial intensity of the Gaussian and CWGB alongside each other in Fig. 2(c)(iii). The lateral waist and axial depth of the beam is quantified by measuring the full width half maximum (FWHM_x_ and FWHM_z_) of the intensity using nonlinear curve fitting. The axial beam depth of focus for the Gaussian beam underfilling lens L1, defined as the FWHM_z_ in the axial direction, was found to be 21 +/− 0.7 µm. This was consistent with an effective lens L1 NA of 0.196, which matches the underfilling of the objective lens. The conical phase (d_c_ = 0.12) generated an extended constant axial intensity of 100 +/− 0.7 µm, experimentally demonstrating the elongated axial intensity of the CWGB. The 4-fold increase in FWHM_z_ in experiment instead of 2.2 folds in the simulation can be attributed to non-paraxial effects that are not taken into account in the thin lens numerical model. However, that said, the marked increase of the FWHM_z_ of the CWGB of a converging lens is consistently observed.

### Back focal plane interferometry using conical wavefront beam (CWGB)

**Figure 3:**
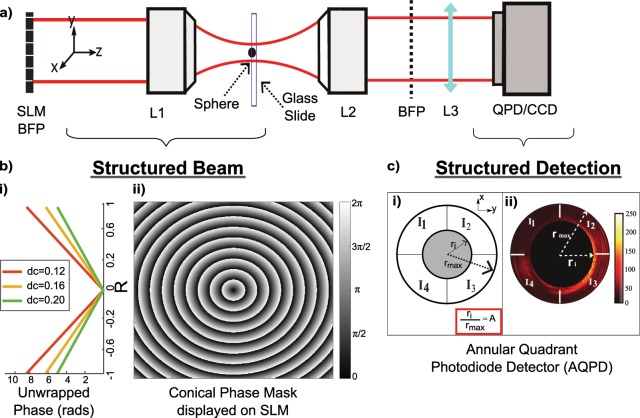
Experimental setup of SBFPI. (**a**) Shows the experimental setup for SBFPI. An SLM is placed at the back focal plane of the focusing microscope objective L1, allowing us to modulate the conical wavefront delay. Another objective L2 is used to image the light, and the relay lens L3 is used to conjugate the BFPI signal to the detector. The detector is a CCD camera operating as an Annular Quadrant Photodiode Detector (APQD) via pixel binning. (**b**) The 2D plot illustrates an example of the (wrapped) phase profile placed onto the SLM to generate the required conical wavefront. The plot to the left of the phase profile shows examples of the unwrapped phase map for different d_c_ parameters. (**c**)(i) Shows a schematic diagram of a BFPI image taken from the camera under annular QPD detection (AQPD) with varying width characterized by A = r_i_/r_max_. In (**c**)(ii), the use of a camera to achieve AQPD operation via pixel binning is shown, along with an example of a back focal plane interference pattern.

Since there is minimal BFPI signal for the QBB as a result of the minimal Gouy phase shift as shown in Fig. 2(a)(v), we focus our investigation on the performance of BFPI by comparing the performance using a Gaussian beam and CWGBs using the experimental setup in Fig. 3(a). We generated CWGBs with different conical wavefronts (d_c_ ranging from 0.10 to 0.20) as shown in Fig. 3(b)(i) using phase masks placed on an SLM as in Fig. 3(b)(ii). Similar to Martinez *et al*.^3^, the detection beam is introduced into the objective lens with low (NA) where the arrangement allows one to achieve extended position detection. We used 3 µm diameter microspheres to generate scattered light that is collected by the higher NA lens L2. The collected scattered and unscattered light constructively interfere at the back focal plane of the collection lens to generate the BFPI signal at the plane of the CCD, which is digitally processed as an Annular QPD (AQPD) as shown in Fig. 3(c).

The beam was first collimated and expanded by a factor of 8.5 to overfill the active area of the SLM (Meadowlark Optics, 7.68 × 7.68 mm, incident angle ≈ 18°). A custom program (MathWorks) projected a blazed hologram of the equivalent wrapped conical phase distribution ($\varnothing =2{\rm{\pi }}R/{d}_{c}$) via the DVI output of a standard personal computer (DELL Optiplex 9010) as in Fig. 3(b) where the wrapped version of b) i) is generated as a 2D phase mask as in b) ii). Here *R* on the SLM phase mask is defined from [−1, 1] which is a unit circle that is matched to the aperture diameter of objective lens of diameter (d = 9.22 mm). In practice, since the SLM normalizes to a unit circle, this means the d_c_ is labeled as a unit of radians^−1^. The SLM is conjugated to the back focal plane of the focusing objective (Obj1, N.A 0.3, 10x) by a 4 f lens system. An iris was placed at the Fourier plane to select only the first diffraction order of the blazed hologram. This setup allows for the removal or introduction of any spatial aberrations, projects a series of conical wavefronts of different d_c_, and permits remote 3D steering of the beam onto samples at nanometric precision^26^ to map the BFPI pattern. The BFPI signal converging at the back focal plane of a second higher NA microscope objective (Obj2, N.A. 0.8, 40x) was conjugated onto a digital camera (Pixelfly USB, PCO) using a 30 mm focal length lens. This long working distance objective, which was selected for ease of measurement^27^, has been previously been demonstrated to create a 3D trap^28^ with stiffness of around 0.03–0.04 pN/µm.

Next, we created the BFPI pattern by air-drying polymer microspheres (3 µm diameter, n = 1.59, Polysciences Inc) bonded via Van Der Waals forces to the surfaces of clear microscope slides. We moved these around the focus position of the beam using a XYZ translation stage (XYFM1, DDSM100/M Thorlabs). For each measurement, the microspheres were positioned as close as possible to the focus of the beam and guided by a viewing camera placed at the image plane instead of the back focal plane. After setting the position of the beam close to a single microsphere, we then remotely translate the beam around the microsphere to map the BFP signal with lateral and axial movement (~363 nm lateral step sizes, ~1 µm axial step). The approach to calibration of BFPI is commonly used when remote beam scanning is available^24^, which is what the SLM offers.

A high-speed camera device (CCD) is used instead of a traditional Quadrant Photodiode Detector (QPD) so as to provide direct visualization of the BFPI pattern with different types of annular rings (A)^3^. Pixel binning is applied to the camera image to obtain the AQPD distributions as shown in Fig. 3(c)(i,ii). This approach provided greater flexibility in identifying and optimizing the SBFPI pattern compared to physical apertures with finite dimensions^8^. The recorded intensity signal is digitized by the camera into 14 bits (2^14^) electrons for each pixel based on the illuminating intensity. Since the normalized signal is typically used for differential detection in a QPD system, we simply refer to the intensity as signal (S). This signal is equal to the total counts registered on the camera.

Lateral displacement is detected by taking the differential signals from opposing sides of the quadrants ${S}_{xy}=\frac{{I}_{1}+{I}_{4}-{I}_{2}-{I}_{3}}{{I}_{total}}$ while axial position detection is conducted by recording the total incident intensity on the position detector ${S}_{z}=\frac{{I}_{1}+{I}_{2}+{I}_{3}+{I}_{4}}{{I}_{total}}$^14^. By this definition both S_xy_ and S_z_ have dimensionless units. Figure 3(c)(i,ii) shows the separation of the four quadrants on the camera I_1_, I_2_, I_3_, I_4_. The dimensions of the annular width at the BFP are identified by the ratio of the inner radius (r_i_) divided by maximum radius of the QPD (r_max_), which we define as A. For SBFPI mode, we analyzed the signals S_xy_ and S_z_ within the annular ring as shown in Fig. 3(c)(ii), while the traditional BFPI results based on the QPD were analyzed based on parameter A = 0 (ie no central block) which we will refer to in the text as AQPD A = 0. All experimental data captured in this paper used a camera and the pixel binning approach described above. From here on, we shall refer to SBFPI for most of the experiments to avoid confusion with BFPI.

### Extended axial detection

We used the experimental setup constructed in the previous section to analyze the performance of SBFPI on the axial signal S_z_ = $\frac{{I}_{{\rm{BFPz}}}}{{I}_{{\rm{total}}}}$ for a range of Δz. A motorized stage moved the bead on the glass slide to known axial positions Δz and tabulated the corresponding axial BFPI signal (normalized sum of the four quadrants of the measurement scheme). In Fig. 3(a)(i) we studied the effects of the CWGB of various d_c_ with AQPD A = 0. This allowed us to study the effect of the CWGB on BFPI performance. In Fig. 3(a)(ii) we studied the effects of various AQPD detection (A = 0 to 0.9) with a fixed CWGB d_c_ = 0.16 rad^−1^. This allowed us to look at the effect of the detection parameter on SBFPI performance.

**Figure 4:**
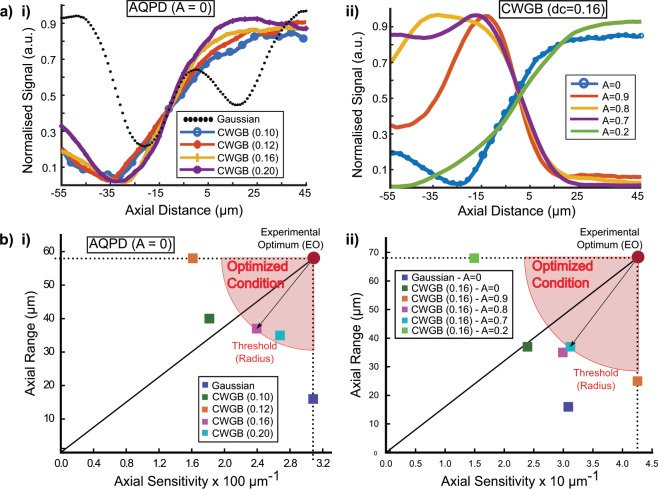
Experimentally obtained SBFPI signal calibration curves with 3 µm polystyrene bead. (**a**)(i) Normalised BFPI signal using AQPD A = 0 against bead axial position for Gaussian beam (control) & various CWGB (d_c_ = 0.10, 0.12, 0.16, 0.20 rad^−1^). (ii) Normalised BFPI signal using CWGB (d_c_ = 0.16 rad^−1^) against axial bead position for various AQPD A parameter values (0, 0.2, 0.7, 0.8, 0.9). (**b**)(i) and (ii) the axial range and sensitivity values of the curves in (**a**)(i) and (ii) respectively are plotted as scatter points. The horizontal axis is the sensitivity (gradient of the linear region), while the vertical axis is the range (horizontal length of the linear region) – please refer to Fig. 1 for information on these parameters. The maximal range and sensitivity observed are plotted as the Experimental Optimum (EO). The distance of each data point from the EO serves as a figure of merit (the lower the better). An example of a threshold distance is shown as a red circle centred about the EO. All points on the circumference of the circle have equal merit with trade-offs between sensitivity and radius. Those closer to the EO are considered to have higher performance.

Figure 4(a)(i) plots the normalized axial displacement signal for Gaussian and various CWGBs with conical phases (∅) from d_c_ = 0.10 rad^−1^ to d_c_ = 0.20 rad^−1^ with AQPD A = 0 detection. The normalized signal is found by first finding the global minimum (GMin) and global maximum (GMax) of all curves on the plot. The GMin is first subtracted from the curves and then divided by (GMax – Gmin). The linear range (ie the number of points comprising the linear region) was calculated through linear fit (*ax + b*) using a linear least squares method in the Matlab Curve Fitting Toolbox, with a goodness of fit threshold of R^2^ = 0.97. For example, for the axial signal curve presented in Fig. 4(a)(i)(orange, d_c_ = 0.16 rad^−1^, AQPD A = 0) we obtained a gradient (sensitivity) of 0.02393 (µm^−1^) and a corresponding linear range of 37 µm with R^2^ = 0.9811 through this method. From this graph, CWGB is experimentally shown to extend the BFPI axial detection range by over 360% from 16 to 58 µm (d_c_ = 0.12 rad^−1^). However, the range extension is observed to reduce the detection sensitivity by over 47% from 0.03083 µm^−1^ to 0.01613 µm^−1^, which can be attributed to the change in beam waist. One notable plot in a) i) is the extended oscillation of the Gaussian beam which interestingly occurs after the linear region. This nonlinear response could be attributed to phase oscillations as a result of the particle itself^29^. While this effect is interesting, the nonlinear response occurring beyond the linear range is not used for BFPI particle tracking and therefore not within the focus of this study.

Across the d_c_ values, we can see that the range and sensitivity vary significantly relative to each other. We generated a scatter plot of the range and the absolute value of the sensitivity to view these changes across the d_c_ variable as shown in Fig. 4(b)(i). We first found the highest range and the highest sensitivity and referred to this as the experimental optimum (EO), a point of reference based on our results. We generated a line from the origin to this point and calculated the distance of each data point form this experimental optimum as a figure of merit (a lower distance indicates a higher improvement). Since this is essentially a distance metric, points that fell on the circumference of a circle centred about the EO are considered equal in performance. The plotted line, however, separates the data points into those which favour range improvement at the expense of sensitivity (above line), and those which favour sensitivity (below line). For example, the CWGB with d_c_ = 0.16 rad^−1^ and d_c_ = 0.20 rad^−1^ fell within the optimized condition circle, but the former was preferred as the AQPD (A = 0) detection range increased 2.31-fold, concomitant with only a mid-range 22.4% reduction in sensitivity. This is in comparison with the d_c_ = 0.20 rad^−1^ data point which had a slightly lower axial range and a slightly higher sensitivity (further away from the line).

We next investigated further extension of the detection range by using AQPD detection^23^ as shown in Fig. 4(a)(ii). The data points were also plotted in (b)(ii) in the same manner as (b)(i), and this point is closest to the EO. In this case the data point closest to the EO is the A = 0.7 case, and the data point is close to the dividing line indicating a reasonable balance between range and sensitivity improvement. Analysing the raw parameters, the axial range is increased 2.31-fold with a 1.2% increase in sensitivity.

### Extended lateral detection

To ensure the completeness of our study, we also investigated the influence of the lateral displacement using the AQPD as in^23^. As mentioned previously, the incident beam was translated across the bead using the SLM as in^24^ which is a commonly used technique when remote beam steering is available. This was done by applying a tilt mask to the SLM in addition to the conical phase, which served to translate the focus of the beam. As the SLM is conjugated to the back focal plane of the condenser objective, the BPFI signal is equivalent to that obtained with a movement of the sample.

**Figure 5:**
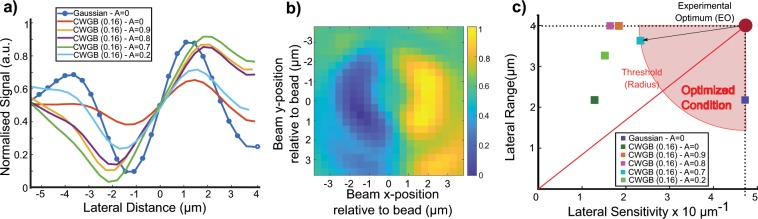
CWGB lateral linearity plots and effects of spatial aberrations on S-BPFI. (**a**) CWGB beam is translated relative to the microsphere along the lateral direction (x, y). Normalized lateral BFPI signal of Gaussian and CWGB (d_c_) against bead lateral position for various AQPD A parameter values (0, 0.9, 0.8, 0.7, 0.2) (**b**) Lateral 2D BFPI signal achieved using CWGB (d_c_ = 0.16 rad^−1^) and A = 0.7. (**c**) Comparison scatter plot of lateral range and sensitivity with varying sizes of annular detection. Note that there are two points that fall within the optimized criterion circle – the Gaussian QPD and the CWGB d_c_ = 0.16 rad^−1^ and AQPD A = 0.7. The latter is the optimal from the dataset given its significant improvement in lateral range.

Figure 5(a) plots the sensitivity and range with varying annular QPD of different A ratios with relative movement of the beam focus against the bead. The raw plots were normalized, and curve fitting conducted based on the same procedure as in the previous section. Based on an annular ratio of 0.7, we quantified the extension of the lateral range as 1.67-fold with a 51% reduction in sensitivity. Figure 5(b) shows a complete 2-dimensional lateral BFPI signal using the annular QPD detection A = 0.7 with a CWGB of conical phase d_c_ of 0.16 rad^−1^. Each pixel corresponds to a particular beam displacement in x and y (bead position at (0,0)), with the intensity displaying the BFPI lateral signal. The 2-D plots matches the expected lateral displacement plots with previous BFPI methods^30^. For reference, a y = 0 horizontal slice of the plot gives the CWGB (d_c_ = 0.16 rad^−1^, A = 0.7) curve in Fig. 5(a)(i).

In Fig. 5(c), we also plot the sensitivity and range of each of the data sets in Fig. 5(a)(i) as we did for the axial BFPI data set in Fig. 4(b). The Gaussian data set with A = 0 displays the greatest sensitivity with a low range. Interestingly, the Gaussian A = 0 data is closest to the EO. We can see overall that the CWGBs generally extend range but result in a loss of sensitivity compared to the Gaussian. For example, the CWGB with A = 0 drastically produces the lowest sensitivity with only a marginal increase in range. The use of an AQPD with nonzero A values (blocking larger proportion of the central part of the BFPI pattern) recovers sensitivity and even significantly increases range. From the CWGB data, the CWGB with A = 0.7 is closest to the EO and hence produces the best experimental result.

### Resilience to spatial aberrations

There has been little attention paid to tackle the robustness of BFPI against aberrations^24^ which is crucial for precise force measurements. All optical microscopy techniques are highly susceptible to spatial optical aberrations because of sample induced refractive index mismatches. And in BFPI, spatial aberrations induce crosstalk between orthogonal position measurements^24^, a potential problem for CWGB beams which could be more easily distorted by refractive index mismatch. To investigate the effects of spatial aberrations in the CWGB, we quantify the SBPFI using a regular QPD (A = 0) versus an AQPD using known levels of lateral aberrations generated by the SLM. After correcting for the system aberrations as in^31^, we then generated various astigmatism phase masks using the astigmatism Zernike polynomial. Astigmatism (much like Coma^24^) is chosen here because of its large asymmetrical intensity distribution that will test the effectiveness of the AQPD.

**Figure 6:**
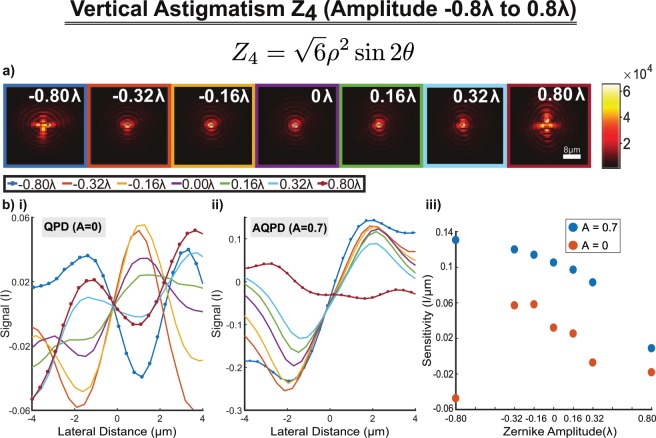
Effect of Aberration (Z4 Astigmatism) of various amplitudes imparted by SLM on Annular QPD detection. (**a**) Intensity profiles at the focus with first order astigmatism (vertical) from −0.8 λ to 0.8 λ (Scale bar is 8 µm). (**b**) Lateral displacement of 3 µm bead against position signal with aberrated CWGB (d_c_ = 0.16 rad^−1^) that is (i) detected through AQPD (A = 0) detection and (ii) AQPD (A = 0.7). The legend is above and common to both plots. iii) Scatter plot of the Sensitivity and Lateral Range extracted from plots (i)(red) and (ii)(blue). These show the average behavior of different AQPD detection, and a clear trend is seen with the A = 0.7 data displaying higher sensitivity than the A = 0.

Figure 6(a) shows the 2D lateral intensity profile of the CWGB after going through a series of vertical astigmatism distortions (−0.8 to 0.8 λ). The asymmetrical intensity distortion along the horizontal and vertical axes can be seen, especially in comparing plots of the same magnitude by opposite sign. Figure 6(b)(i,ii) plot the lateral displacement signal against lateral translations of a 3 µm bead for QPD (A = 0) and AQPD (A = 0.7) detection respectively. Each curve corresponds to a different amplitude of the Z_4_ astigmatism phase mask for which the accompanying beam spot is provided in a). From the SBFPI plots, it appears that the linearity curves with the AQPD displayed minimal loss of sensitivity as compared to traditional QPD. From a visual observation, the signal calibration curves for A = 0.7 in ii) remain ordered for almost all astigmatism amplitudes, unlike those in i) which become relatively disordered (ie the characteristic linear region is missing or distorted). Upon further inspection, the A = 0 situation is resilient to Z_4_ amplitudes of −0.32 λ, −0.16 λ, 0λ, but affected by all others (even those of same magnitude by opposite sign). One reason for this is the asymmetrical intensity distortion, which explains why both magnitude and sign are important. Notably the A = 0.7 situation is only significantly affected by this asymmetry for the +0.80λ amplitude (ie the signal curve cannot be used for SBFPI).

In order to compare the benefits of AQPD detection, we plotted the sensitivity of each plot against the applied Zernike amplitude in Fig. 6(b)(iii). We plotted the A = 0 data in red, and the A = 0.7 data in blue, allowing us to observe systematic trends in the data. We can see that the sensitivity of the A = 0.7 data is consistently higher than the A = 0, once again demonstrating the resilience to spatial aberration as seen in b) i) and ii). This resilience to spatial aberrations in AQPD is likely because the central intensity of the SBFPI, zero order interference pattern which contains a large component of unscattered light is blocked.

## Conclusion

In conclusion, the new SBFPI technique validates that a CWGB possesses an extended depth of focus whilst retaining the important axial phase shift (Gouy phase) and minimal change of lateral beam waist. Unlike Bessel beams which rely on an annular intensity ring in the back focal plane, the CWGB relies on a smooth conical wavefront while maintaining the Gaussian intensity, producing different effects at the focus. Together with complimentary AQPD detection, we found that the experimental datasets at the Experimental Optimum have the capability to enhance axial (4.25-fold) and lateral (1.83-fold) displacement tracking range, although these have sensitivity drops of 53% and 61% respectively. This result was obtained without requiring additional beam scanners, nonlinear calibration or holographic detections. The combination of CWGBs and AQPDs allows one to fine tune the tracking range and sensitivity by adjusting the AQPD annular ring diameter and CWGB depth of focus, resulting in a range of linearity curves^23^ over the same particle size. This could be advantageous in tracking non-spherical particles such as nanorods and nanowires^20^, as tracking parameters can be tailored on the fly to individual experiments. Furthermore, SBFPI was found to be more resilient to spatial aberrations compared to traditional Gaussian BFPI. SBFPI can be easily implemented on existing BFPI systems - the axial depth of conical wavefront beams can be varied by changing the beam waist of the Gaussian beam inserted into a simple holographic mask or conical phase element conjugate to the back pupil plane, while the annular detection is a simple modification to existing quadrant photodetectors^23^. Moreover, the use of a CWGB could be beneficial to other fields of research that require an extended depth of field while maintaining beam waist, as in^32,33^.

## References

[1] Pralle, A., Prummer, M., Florin, E.-L., Stelzer, E. H. K. & Hörber, J. K. H. Three-dimensional high-resolution particle tracking for optical tweezers by forward scattered light. *Microscopy Research and Technique* **44**, 378–386, doi:10.1002/(sici)1097-0029(19990301)44:5<378::Aid-jemt10>3.0.Co;2-z (1999). 10.1002/(SICI)1097-0029(19990301)44:5<378::AID-JEMT10>3.0.CO;2-Z 10090214

[2] Rohrbach A, Kress H, Stelzer EHK (2003). Three-dimensional tracking of small spheres in focused laser beams: influence of the detection angular aperture. Opt. Lett..

[3] Martínez IA, Petrov D (2012). Back-focal-plane position detection with extended linear range for photonic force microscopy. Appl. Opt..

[4] Rohrbach A, Stelzer EH (2002). Three-dimensional position detection of optically trapped dielectric particles. Journal of Applied Physics.

[5] Perrone S, Volpe G, Petrov D (2008). 10-fold detection range increase in quadrant-photodiode position sensing for photonic force microscope. Review of Scientific Instruments.

[6] Gittes F, Schmidt CF (1998). Interference model for back-focal-plane displacement detection in optical tweezers. Opt. Lett..

[7] Allersma MW, Gittes F, deCastro MJ, Stewart RJ, Schmidt CF (1998). Two-Dimensional Tracking of ncd Motility by Back Focal Plane Interferometry. Biophysical Journal.

[8] Faegheh H, Mousavi SM, Zeinab SK, Reihani SNS (2014). Extended linear detection range for optical tweezers using image-plane detection scheme. Journal of Optics.

[9] Taylor MA, Knittel J, Hsu MTL, Bachor HA, Bowen WP (2011). Sagnac interferometer-enhanced particle tracking in optical tweezers. Journal of Optics.

[10] Phillips DB (2011). Surface imaging using holographic optical tweezers. Nanotechnology.

[11] Denk W, Webb WW (1990). Optical measurement of picometer displacements of transparent microscopic objects. Appl. Opt..

[12] King GM, Carter AR, Churnside AB, Eberle LS, Perkins TT (2009). Ultrastable atomic force microscopy: atomic-scale stability and registration in ambient conditions. Nano letters.

[13] Moffitt JR, Chemla YR, Izhaky D, Bustamante C (2006). Differential detection of dual traps improves the spatial resolution of optical tweezers. Proceedings of the National Academy of Sciences.

[14] Neuman KC, Nagy A (2008). Single-molecule force spectroscopy: optical tweezers, magnetic tweezers and atomic force microscopy. Nature Methods.

[15] Neuman KC, Block SM (2004). Optical trapping. Review of scientific instruments.

[16] Friedrich L, Rohrbach A (2012). Tuning the detection sensitivity: a model for axial backfocal plane interferometric tracking. Opt. Lett..

[17] Friedrich L, Rohrbach A (2010). Improved interferometric tracking of trapped particles using two frequency-detuned beams. Opt. Lett..

[18] Kim M-S (2012). Phase anomalies in Bessel-Gauss beams. Opt. Express.

[19] Jannasch A, Demirörs AF, van Oostrum PDJ, van Blaaderen A, Schäffer E (2012). Nanonewton optical force trap employing anti-reflection coated, high-refractive-index titania microspheres. Nature Photonics.

[20] Pauzauskie PJ (2006). Optical trapping and integration of semiconductor nanowire assemblies in water. Nature Materials.

[21] Ayala YA, Arzola AV, Volke-Sepúlveda K (2016). 3D micromanipulation at low numerical aperture with a single light beam: the focused-Bessel trap. Opt. Lett..

[22] Deufel C, Wang MD (2006). Detection of Forces and Displacements along the Axial Direction in an Optical Trap. Biophysical Journal.

[23] Mousavi SM, Akbar S, Faegheh H, Reihani SNS (2015). Extended linear detection range for optical tweezers using a stop at the back focal plane of the condenser. Journal of Optics.

[24] Dixon TF, Russell LW, Andres-Arroyo A, Reece PJ (2017). Using back focal plane interferometry to probe the influence of Zernike aberrations in optical tweezers. Opt. Lett..

[25] Sheppard CJR, Wilson T (1978). Gaussian-beam theory of lenses with annular aperture. IEE Journal on Microwaves, Optics and Acoustics.

[26] Schmitz CHJ, Spatz JP, Curtis JE (2005). High-precision steering of multiple holographic optical traps. Opt. Express.

[27] Rodrigues VRM (2013). Enhancing the Strength of an Optical Trap by Truncation. PLOS ONE.

[28] Samadi A, Reihani NS (2010). Optimal beam diameter for optical tweezers. Opt. Lett..

[29] Hwang J, Moerner WE (2007). Interferometry of a single nanoparticle using the Gouy phase of a focused laser beam. Optics Communications.

[30] Koch, M. D. & Shaevitz, J. W. In *Optical Tweezers: Methods and Protocols* (ed. Arne Gennerich) 3–24 (Springer New York, 2017).

[31] Zhu W (2016). Dynamic axial control over optically levitating particles in air with an electrically-tunable variable-focus lens. Biomed. Opt. Express.

[32] Dufour P, Piché M, De Koninck Y, McCarthy N (2006). Two-photon excitation fluorescence microscopy with a high depth of field using an axicon. Appl. Opt..

[33] Shechtman Y, Weiss LE, Backer AS, Sahl SJ, Moerner WE (2015). Precise Three-Dimensional Scan-Free Multiple-Particle Tracking over Large Axial Ranges with Tetrapod Point Spread Functions. Nano Letters.

